# Targeting Chaperone/Co-Chaperone Interactions with Small Molecules: A Novel Approach to Tackle Neurodegenerative Diseases

**DOI:** 10.3390/cells10102596

**Published:** 2021-09-29

**Authors:** Lisha Wang, Liza Bergkvist, Rajnish Kumar, Bengt Winblad, Pavel F. Pavlov

**Affiliations:** 1 Department of Neuroscience Care and Society, Division of Neurogeriatrics, Karolinska Institutet, 17164 Solna, Sweden; lisha.wang@ki.se (L.W.); lizabergkvist@gmail.com (L.B.); rajnish.kumar@ki.se (R.K.); bengt.winblad@ki.se (B.W.); 2 Department of Pharmaceutical Engineering & Technology, Indian Institute of Technology (BHU), Varanasi 221005, India; 3 Theme Inflammation and Aging, Karolinska University Hospital, 14186 Huddinge, Sweden

**Keywords:** Hsp70, Hsp90, co-chaperones, neurodegenerative diseases, small molecules

## Abstract

The dysfunction of the proteostasis network is a molecular hallmark of neurodegenerative diseases such as Alzheimer’s disease, Parkinson’s disease, Huntington’s disease, and amyotrophic lateral sclerosis. Molecular chaperones are a major component of the proteostasis network and maintain cellular homeostasis by folding client proteins, assisting with intracellular transport, and interfering with protein aggregation or degradation. Heat shock protein 70 kDa (Hsp70) and 90 kDa (Hsp90) are two of the most important chaperones whose functions are dependent on ATP hydrolysis and collaboration with their co-chaperones. Numerous studies implicate Hsp70, Hsp90, and their co-chaperones in neurodegenerative diseases. Targeting the specific protein–protein interactions between chaperones and their particular partner co-chaperones with small molecules provides an opportunity to specifically modulate Hsp70 or Hsp90 function for neurodegenerative diseases. Here, we review the roles of co-chaperones in Hsp70 or Hsp90 chaperone cycles, the impacts of co-chaperones in neurodegenerative diseases, and the development of small molecules modulating chaperone/co-chaperone interactions. We also provide a future perspective of drug development targeting chaperone/co-chaperone interactions for neurodegenerative diseases.

## 1. Introduction

Proteins perform different functions that are essential for the physiology of an organism. The proteostasis network maintains the health of the proteome by controlling the protein synthesis, folding, trafficking, disaggregation, and degradation [1]. The dysfunction of the proteostasis network is a hallmark of neurodegenerative diseases, such as Alzheimer’s disease (AD), Parkinson’s disease (PD), Huntington’s disease (HD), and amyotrophic lateral sclerosis (ALS) [2]. Molecular chaperones play an important role in the proteostasis network. They maintain cellular homeostasis by folding client proteins, assisting with intracellular transport, interfering with protein aggregation, or directing misfolded or aggregated proteins to cellular clearance pathways. Heat shock protein 70 kDa (Hsp70) and 90 kDa (Hsp90) are two of the most important chaperone families whose functions are dependent on ATP hydrolysis to provide energy and collaboration with their co-chaperones to form the dynamic complex. Healthy proteostasis is especially important for virtually non-dividing neurons in the adult brain; it is not surprising that many studies have implicated Hsp70, Hsp90, and their co-chaperones in neurodegenerative diseases [3,4,5]. Modulation of the functions of Hsp70 and Hsp90 has been regarded as an attractive pharmacological intervention for neurodegenerative diseases.

Although much work has gone into the development of Hsp70 or Hsp90 inhibitors in cancer, none of them have had success in clinical trials owing to their adverse effects. Most inhibitors were discovered to completely inhibit chaperone functions by occupying the N-terminal ATP binding pocket [6,7]. However, Hsp70 and Hsp90 regulate a large spectrum of client proteins and inhibiting ATPase function can also interfere with all of their client proteins, leading to undesirable/toxic effects, making this an unsuccessful strategy. Moreover, the inhibition of Hsp90 ATPase activity triggers a compensatory mechanism resulting in a heat shock response [8]. Another option is to target the Hsp90 C-terminus, which does not induce detrimental heat shock response. The C-terminal inhibitors have been well-summarized in recent reviews and employ multiple mechanisms including inhibition of C-terminal dimerization, induction of chaperone conformational cycles, and modulation of the interaction with co-chaperones [9,10]. Among them, dihydropyridine derivatives have shown neuroprotective effects *in vivo* [11,12]. However, this approach has been limited owing to the lack of complete structural information and exact binding sites in the C-terminus [13].

Therefore, alternative approaches to modulate Hsp70 or Hsp90 are needed. Numerous co-chaperones associate with Hsp70 or Hsp90 at different stages of chaperone cycles and appear to be dependent on the substrates involved in neurodegenerative diseases. Targeting the protein–protein interactions (PPIs) between chaperones and their particular partner co-chaperones provides the opportunity to specifically modulate Hsp70 or Hsp90 function in neurodegenerative diseases. In this review, we summarize the knowledge of the involvement of co-chaperones in Hsp70 or Hsp90 chaperone cycles, the functions of co-chaperones in neurodegenerative diseases, and the development of small molecules that modulate chaperone/co-chaperone PPIs. We also discuss the dicarboxylate clamp mechanism in the interactions of Hsp90 C-terminal Met-Glu-Glu-Val-Asp (MEEVD) peptide and tetratricopeptide repeat (TPR) domain of co-chaperones and provide useful insights for future drug development targeting chaperone/co-chaperone interactions for neurodegenerative diseases.

## 2. Hsp70 and Its Co-Chaperones

### 2.1. Hsp70 Structure and Chaperone Cycle

**Figure 1:**
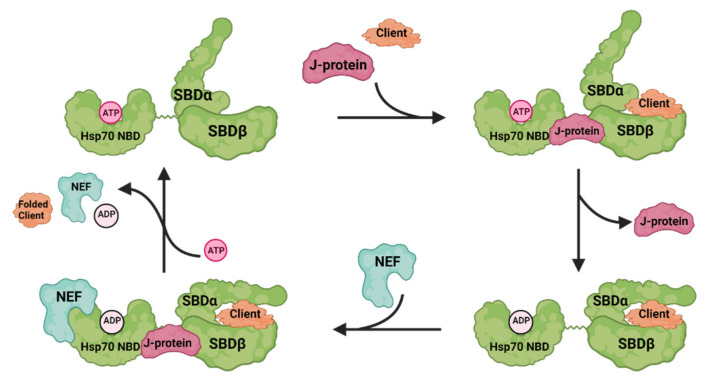
A schematic illustration of the Hsp70 chaperone cycle. Hsp70 consists of two domains connected by a flexible linker: an N-terminal ATP binding domain (NBD) and a C-terminal substrate/client binding domain (SBD), which is divided into a β subdomain (SBDβ), hosting the substrate binding site, and an α subdomain (SBDα), forming a lid lock structure to trap clients. When ATP binds to the NBD, the lid formed by SBDα opens. Co-chaperone J-protein binds to Hsp70 to stimulate its ATPase activity and facilitate client binding in the hydrophobic pocket of SBDβ. Hydrolysis of ATP results in the closing of the C-terminal lid (SBDα) and trapping of the client. Another co-chaperone, nucleotide exchange factor (NEF), binds to the NBD of Hsp70 to accelerate the release of ADP. Then, SBDα opens and the folded client is able to leave Hsp70. Created with BioRender.com.

Hsp70 consists of two domains connected by a flexible linker: an N-terminal ATP binding domain (NBD) and a C-terminal substrate/client binding domain (SBD). SBD is divided into a β subdomain (SBDβ), hosting the substrate binding site, and an α subdomain (SBDα), forming a lid lock structure to trap client proteins. Hsp70 function is dependent on the coordinated activity of all three domains [14] (Figure 1). When ATP binds the NBD, the lid formed by SBDα opens and client proteins are now able to bind in the hydrophobic pocket found in the SBDβ. Hydrolysis of ATP results in the closing of the C-terminal lid (SBDα) and trapping of the client protein. Release of ADP opens the C-terminal lid and client proteins are able to leave the SBD. However, the ability of Hsp70 to hydrolyze ATP is intrinsically low. To facilitate this process, the co-chaperones of Hsp70 step in, such as J-proteins (also called Hsp40s) and nucleotide exchange factors (NEFs). J-proteins all share a 70 amino acid stretch, the J-domain, which interacts with Hsp70 NBD, the inter-domain linker, and SBDβ to stimulate its ATPase activity and facilitate client capture [15,16]. NEFs can be categorized into four distinct families: GrpE, Hsp110, HspBP1, and Bag proteins [17]. They bind to the NBD of Hsp70 to accelerate the release of ADP and, in some cases, foster client dissociation. Humans have 13 Hsp70s, 41 J-proteins, and 13 NEFs, which play a major role in driving the multiple functions of Hsp70 [18]. Taken together, the co-chaperones of Hsp70 are thought to modulate the enzymatic activity of the chaperone and help to guide its interactions with client proteins. Therefore, inhibiting the interaction between Hsp70 and its specific co-chaperones could be a therapeutic strategy to target specific Hsp70 functions, thus avoiding adverse effects arising from the general inhibition of Hsp70 function.

### 2.2. Hsp70 and Its Co-Chaperones in Neurodegenerative Diseases

The role of Hsp70 in protein homeostasis connects it closely to protein misfolding and neurodegenerative diseases like AD, PD, HD, and prion disease [5]. In addition to re-folding misfolded proteins, Hsp70 has been shown to inhibit Aβ aggregation, as well as promote degradation of Aβ and tau aggregates via the proteasomal system [5]. On the contrary, the constitutively expressed Hsc70, one of Hsp70 family members, prevents tau degradation, and an imbalance in the Hsp70 family and their co-chaperones promotes tau pathology [19]. Additional potential pathogenic involvement of Hsp70 in protein misfolding diseases was shown by Fontaine and colleagues: Hsp70 in complex with its co-chaperone J-protein DnaJC5 controls the extracellular release of disease-associated proteins like tau, α-synuclein, and transactive response DNA-binding protein 43 (TDP-43), providing a mechanism for how aggregation-prone proteins are able to leave the cell and possibly propagate [20]. It was recently shown that Hsp70 in complex with its co-chaperones, the J-proteins DNAJB1 and HSPA4 and an Hsp110-type NEF, disassembles tau fibrils *in vitro*, which results in the release of seeding potent oligomeric tau [21]. This suggests that the anti-aggregation properties of Hsp70 are a two-edged sword, as it eliminates tau fibrils at the cost of generating new seeds. Therefore, potential future treatment strategies for neurodegenerative, protein misfolding diseases could be identifying small molecules targeting specific Hsp70 isoforms and their co-chaperones that contribute to pathogenesis.

### 2.3. Hsp70 Co-Chaperone Interaction Inhibitors

**Figure 2:**
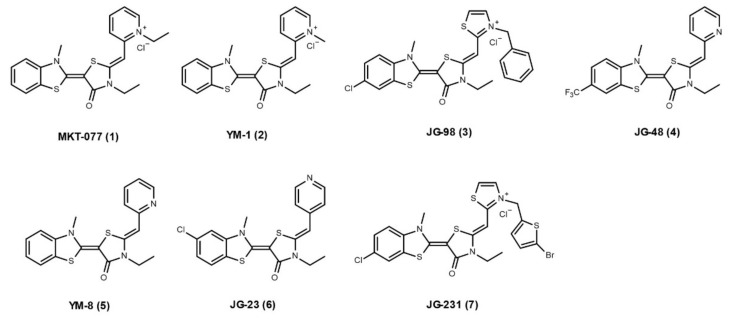
Small molecules with the ability to disrupt Hsp70/co-chaperone interactions.

No specific, competitive inhibitors have been developed for Hsp70 and its co-chaperones. Thus far, the Hsp70 inhibitors explored have either been targeting ATPase activity or resulted in allosteric inhibition. Allosteric inhibitors, i.e., non-competitive inhibitors, of Hsp70 can be divided into three classes [22]. For two of these classes of Hsp70 allosteric inhibitors (exemplified by VER-155008 and YK5), few studies have shown any effect on Hsp70 co-chaperone interactions. The third class of allosteric inhibitors are based on **MKT-077** (**1**, Figure 2) (with next generation molecules like YM-1 and JG-98) and have been shown to impact chaperone/co-chaperone interactions. Structural studies showed that MKT-077 and analogues bind to a highly conserved hydrophobic pocket next to the NBD, with binding being favoured in the ADP-bound state of Hsp70. Although MKT-077 analogues bind >20Å away from the Hsp70–NEF interaction surface, they trap a conformational state that disfavors specific co-chaperone interaction, preventing the NEF family of Bag proteins from binding [22]. **YM-1** (**2**, Figure 2) and **JG-98** (**3**, Figure 2) were both shown to inhibit Hsp70–Bag3 interactions by a pulldown assay, and their treatments are sufficient to suppress tumor growth in mice that mirrors the effects of Hsp70 depletion [23,24].

The role of inhibiting the interactions between Hsp70 and its co-chaperones in the context of neurodegenerative diseases has also been investigated. **JG-48** (**4**, Figure 2) is an analogue of YM-1, developed to not interfere with Hsp70 chaperone activity. Using this compound, Young et al., observed an increase in tau turnover in vitro [25]. JG-48 is partially able to inhibit the NEF family of Bag proteins from interacting with Hsp70. This results in a stabilized Hsp70–tau complex, subsequently initiating degradation of tau. Another YM-1 analogue, **YM-8** (**5**, Figure 2), is able to penetrate the blood–brain barrier and decrease tau phosphorylation in cultured brain slices [26]. In a recent study, Shao et al. further improved the pharmacokinetic properties of YM-8, resulting in the new compound **JG-23** (**6**, Figure 2) [27]. JG-23 is 12 times more stable than YM-8, while still retaining promoting tau degradation *in vitro*.

Other MKT-077 analogues, e.g., **JG-231** (**7**, Figure 2), have also been shown to inhibit the interaction between Hsp70 and J-proteins. In a recent study by Bengoechea and colleagues, they showed that both genetical and pharmacological inhibition (JG-231) of the interaction between Hsp70 and DnaJB6 was beneficial in a mouse model of muscular dystrophy [28]. However, none of these allosteric inhibitors have been translated into the clinic; only MKT-077 has been tested in a clinical trial setting, where it failed owing to renal toxicity in phase I.

## 3. Hsp90 and Its Co-Chaperones

### 3.1. Hsp90 Structure and Chaperone Cycles

Hsp90 serves as a platform for folding and maturation of many client proteins, such as steroid hormone receptors (SHRs), protein kinases, transcription factors, and E3 ubiquitin ligases. To fulfil its function, Hsp90 homodimer undergoes ATP-regulated conformational rearrangements. Hsp90 is composed of three domains: the N-terminal domain (NTD), the middle domain (MD), and the C-terminal domain (CTD). The Hsp90 NTD has the ATP binding pocket, the MD is important for ATP hydrolysis and binding with the client proteins, while the CTD ends in an MEEVD motif that is responsible for interacting with the TPR domains present in a subgroup of Hsp90 co-chaperones [29]. In the absence of ATP, Hsp90 homodimer adopts an open V-shaped conformation. Upon ATP binding to the NTD, the N-terminal lids close over the bound ATP, leading to the intermediate state. Further structural rearrangements induce the NTD dimerize to form the closed 1 state and then associate with the MD to form the closed 2 state. After ATP hydrolysis by the residues from Hsp90 MD, Hsp90 returns to the open conformation and ADP and inorganic phosphate (Pi) are released.

**Figure 3:**
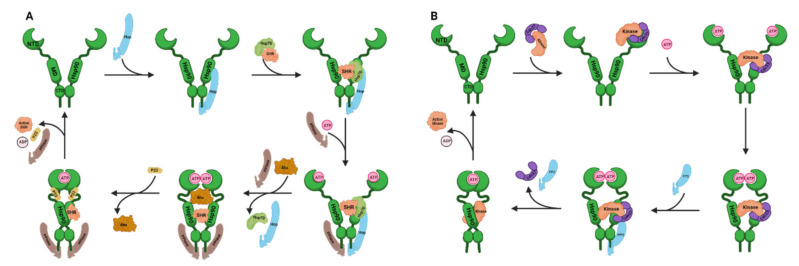
(**A**) Schematic illustration of Hsp90 chaperone cycles. Hsp90 includes three domains: the N-terminal domain (NTD), the middle domain (MD), and the C-terminal domain (CTD). Hsp90 homodimer undergoes ATP-regulated conformational rearrangements. (**A**) Hsp90-driven activation of steroid hormone receptors (SHRs). Hsp70–Hsp90 organizing protein (Hop, also known as stress-inducible phosphoprotein 1, Sti1) transfers unfolded SHRs from Hsp70 to Hsp90 and binds to one of the tetratricopeptide repeat (TPR)-acceptor sites of Hsp90 in the open state. The co-chaperone with peptidyl-prolyl cis-trans isomerase (PPIase) domain binds to the other Hsp90 TPR-acceptor site to form an asymmetric complex, and the binding of ATP leads to the Hsp90 intermediate state. Activator of Hsp90 ATPase homolog 1 (Aha1) promotes the formation of Hsp90 closed 1 state and accelerates ATPase activity. Co-chaperone p23 competes with Aha1 for binding to Hsp90 and stabilizes Hsp90 closed 2 state by reducing ATPase activity. After ATP hydrolysis, p23, the PPIase, and the active SHR are released. (**B**) Hsp90-driven activation of the protein kinases. After phosphorylation, Cdc37 captures the kinase to form a Cdc37–kinase complex that binds to Hsp90 NTD. In the presence of ATP, the Cdc37–kinase complex moves to Hsp90 MD, and Hsp90 transforms from an open state to a closed state. Then, protein phosphatase 5 (PP5) binds to Hsp90 CTD and dephosphorylates Cdc37. Finally, Cdc37, PP5, and active kinase are released, and Hsp90 returns to the open state for another cycle. Created with BioRender.com.

The dynamic conformational changes of Hsp90 are regulated by a set of Hsp90 co-chaperones (Table 1). Complex formation between Hsp90 and its clients has been studied most extensively for SHRs [30] (Figure 3A). At first, Hsp70–Hsp90 organizing protein (Hop, also known as stress-inducible phosphoprotein 1, Sti1) facilitates unfolded SHRs transferred from Hsp70 to Hsp90 and binds to the open conformation of Hsp90 and inhibits Hsp90 ATPase activity. The co-chaperone with a peptidyl-prolyl *cis*-*trans* isomerase (PPIase) domain, like the FK506 binding protein (FKBP) 51 or FKBP52, binds to the other Hsp90 TPR-acceptor site to form an asymmetric complex. The binding of ATP results in the intermediate state of Hsp90. Activator of Hsp90 ATPase homolog 1 (Aha1) promotes the formation of the closed 1 state and accelerates Hsp90 ATPase activity. Co-chaperone p23 (Sba1 in yeast) competes with Aha1 for binding to Hsp90 and stabilizes the closed 2 state by reducing Hsp90 ATPase activity. After ATP hydrolysis, p23, the PPIase, and the folded client are released from Hsp90. For protein kinases, the Hsp90 cycle (Figure 3B) is described specifically below (see Section 3.2.1). Cell division cycle 37 (Cdc37) recognizes and delivers an extensive range of protein kinases to Hsp90, which meditates kinase maturation. Protein phosphatase 5 (PP5, also known as Ppt1 in yeast) dephosphorylates Cdc37 at the late stage, leading Hsp90 to adopt an open state and repeat the chaperone cycle.

### 3.2. Hsp90 and Its Co-Chaperones in Neurodegenerative Diseases and Their PPI Inhibitors

Extensive research supports the important roles of Hsp90 co-chaperones in various neurodegenerative diseases, which are summarized in Table 1 and described in the following subsections. Unlike directly inhibiting Hsp90, specifically targeting these Hsp90/co-chaperones PPIs can modulate Hsp90 function by “fine-tuning” and provide an alternative therapeutic strategy for neurodegenerative diseases. Until now, many small molecules have been reported to inhibit the interactions between Hsp90 and Cdc37, Aha1, p23, Hop, or other TPR co-chaperones. The following subsections highlight PPIs of interest and the developed PPI inhibitors, as well as their binding sites, drug development, and chemical structures.

#### 3.2.1. Hsp90–Cdc37 Interaction

Cdc37 specifically recognizes and delivers an extensive range of protein kinases to Hsp90, which forms an Hsp90–Cdc37–kinase complex that mediates kinase maturation [7]. Several lines of evidence prove the roles of the Hsp90–Cdc37 complex in neurodegeneration. Cdc37 stabilizes tau via Hsp90 and regulates the stability of distinct tau kinases, specifically cyclin-dependent kinase 5 (Cdk5) and Akt [31]. The Hsp90–Cdc37 complex also preserves TDP-43, which mislocalizes and accumulates in the cytoplasm in the ALS, frontal temporal dementia (FTLD), and some cases of AD [32]. Moreover, the client kinases of the Hsp90–Cdc37 chaperone system also include dual-specificity tyrosine-phosphorylation-regulated kinase 1A (DYRK1A), leucine-rich repeat kinase 2 (LRRK2), and PTEN-induced kinase 1 (PINK1), which are involved in AD and PD [33,34,35,36]. Unlike directly inhibiting Hsp90 ATPase, blocking the Hsp90–Cdc37–kinase chaperone cycle could selectively modulate Hsp90 kinase clients, rather than inhibiting all the Hsp90 client proteins. Therefore, disrupting Hsp90–Cdc37–kinase interactions has emerged as a potential alternative therapeutic strategy for neurodegenerative diseases.

Two different states of Hsp90–Cdc37 complex structures (PDB: 1US7 and PDB: 5FWL) have been identified by Roe et al., in 2004 and Verba et al., in 2016, respectively [67,68]. These structures provide essential clues to understand the binding between Hsp90 and Cdc37. Before ATP binding, Hsp90 exhibits a clamp-like open state. After phosphorylation by CK2, Cdc37 can capture the client kinase to form a Cdc37–kinase complex that binds to the NTD of Hsp90 via Cdc37 MD. When the NTD of Hsp90 binds ATP, Cdc37-kinase complex moves to the MD of Hsp90, which interacts with the NTD of Cdc37 and the N-lobe of the kinase. Then, Hsp90 transforms from an open state to a closed state. After that, PP5 binds to the CTD of Hsp90 and dephosphorylates Cdc37. Then, Cdc37, PP5, and active kinase are released and Hsp90 returns to the open state for another chaperone cycle [7] (Figure 3B). This Hsp90–Cdc37 binding process makes it possible to discover compounds disrupting Hsp90–Cdc37–kinase interactions.

**Figure 4:**
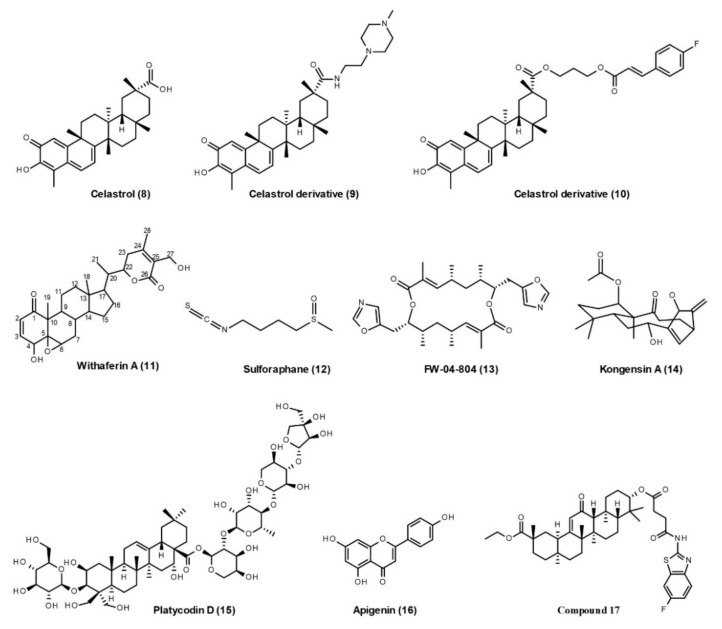
Natural products and their derivatives with the ability to disrupt Hsp90–Cdc37 interactions.

**Figure 5:**
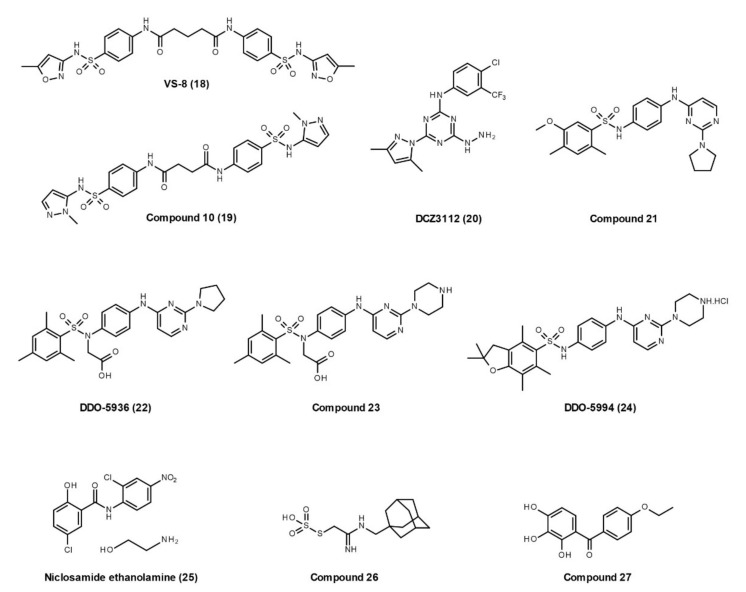
Developed small molecules with the ability to disrupt Hsp90–Cdc37 interactions.

The developed inhibitors targeting Hsp90–Cdc37 interactions include naturally derived products (Figure 4), Cdc37 peptides, and small molecular inhibitors (Figure 5).

##### Natural Products

Some natural products have been found to inhibit the Hsp90–Cdc37 interaction, such as celastrol, withaferin A, sulforaphane, FW-04-806, kongensin A, platycodin D, apigenin, and 18β-glycyrrhetinic acid derivatives. Although these natural products are nonspecific and have poor druggability, their drug-like derivatives could be developed as a therapy for neurodegenerative diseases.

**Celastrol** (**8**, Figure 4) is a quinone methide triterpene isolated from the *Tripterygium wilfordii* Hook F. In pancreatic cancer cells, celastrol disrupts the Hsp90–Cdc37 interaction, but does not disrupt Hsp90–Hop and Hsp90–p23 interactions [69]. Several studies have investigated the binding sites of celastrol on the Hsp90–Cdc37 complex, although with conflicting results. In 2009, proteolytic fingerprinting indicated that celastrol binds to the CTD of Hsp90 to protect it from trypsin digestion [70], while HSQC NMR studies show that the quinone methide of celastrol reacts with the thiol group of Cdc37 in the NTD through a Michael addition, thereby disrupting the Hsp90–Cdc37 complex [71]. In 2014, multiple techniques, such as size-exclusion chromatography coupled to multi-angle laser light scattering, native-PAGE, dynamic light scattering, differential scanning calorimetry, differential scanning fluorescence, and chaperone and PPI assays, have been applied to characterize the interaction, and it was found that celastrol affects the oligomeric state of Hsp90 by binding to its CTD [72]. Thus, the action mode of celastrol is not only via its “Michael acceptor’’ functionality, but also involves other mechanisms that need to be clarified in further studies. To improve the drug-like properties of celastrol, many celastrol derivatives have recently been produced. Two of them (**9** and **10**) have been selected for improved Hsp90–Cdc37 disruption activity and antiproliferative activity [73,74]. Although celastrol has non-negligible anti-tumor efficacy [75], it possesses an extensive medical value in the treatment of neurodegenerative diseases such as AD, PD, ALS, cerebral ischemia, multiple sclerosis, and spinal cord injury, which has been well-summarized in a recently published review [76]. The celastrol derivatives targeting neurodegenerative diseases need to be developed in the future.

**Withaferin A** (**11**, Figure 4) is steroidal lactone isolated from the *Withania somnifera*. In pancreatic cancer cells, withaferin A disrupts the Hsp90–Cdc37 interaction, while it neither blocks ATP binding to Hsp90 nor disrupts the Hsp90–p23 interaction [77]. A withaferin A-biotin pull-down assay shows that Withaferin A binds to the CTD of Hsp90 [77], suggesting that targeting the Hsp90 CTD may disrupt the Hsp90–Cdc37 interaction by allosteric regulation. Later, the computational docking results indicate that withaferin A has the potential to inhibit the Hsp90–Cdc37 interaction by disrupting the stability of the Hsp90–Cdc37 complex [78]. Structure–activity relationship studies show that the C-5(6) epoxy functional group of withaferin A is required for binding with Hsp90, the substitution of C-2,3 position may hinder its inhibition on Hsp90 activity, while the C-4 hydroxyl group in its A ring may enhance the inhibition on Hsp90 and disruption of the Hsp90–Cdc37 complex [79]. Withaferin A is not only a promising anticancer compound, but also has many other therapeutic benefits, including neuroprotective (AD, PD, and ALS), cardioprotective, anti-viral (COVID-19 and Hepatitis), and osteoporotic effects [80].

**Sulforaphane** (**12**, Figure 4) is an isothiocyanate found in cruciferous vegetables, such as cabbage, broccoli, cauliflower, and kale. In pancreatic cancer cells, sulforaphane induces the Hsp90 degradation and blocks the Hsp90–Cdc37 interaction without affecting the ATP binding pocket of Hsp90 [81]. NMR and liquid chromatography coupled to mass spectrometry studies reveal that sulforaphane binds to the NTD of Hsp90 [81]. Sulforaphane has shown efficacy in a wide range of human-related neurological pathologies, such as AD, PD, HD, ALS, multiple sclerosis, autism spectrum disorder, and schizophrenia [82,83]. There is now a clinical study recruiting participants to investigate the effects of sulforaphane in patients with prodromal to mild AD (NCT04213391).

**FW-04-806** (**13**, Figure 4), also known as conglobatin A, is extracted from the China-native *Streptomyces* FIM-04-806. Immunoprecipitation confirms that FW-04-806 disrupts the Hsp90–Cdc37 interaction, leading to enhanced tumor-arresting activity and the degradation of Hsp90 clients in breast cancer cells [84]. Chemoproteomics and computational approaches together reveal that FW-04-806 binds to the NTD of Hsp90 without affecting ATP binding of Hsp90 [84]. Recent computational docking results indicate that FW-04-806 sterically blocks and disturbs critical interactions, notably between Glu47 of Hsp90 NTD and Arg167 of Cdc37 at the interface of the complex (PDB: 1US7) [85].

**Kongensin A** (**14**, Figure 4) is isolated from *Croton kongensis* and has the potential to inhibit necroptosis and induce apoptosis. The bio-orthogonal ligation method reveals that Hsp90 is the direct cellular target of kongensin A, and further studies demonstrate that kongensin A binds covalently to Cys420 on the MD of Hsp90, dissociates Hsp90 from Cdc37, and subsequently inhibits necroptosis [86,87].

**Platycodin D** (**15**, Figure 4) is an effective triterpenesaponin isolated from the roots of *Platycodon grandiflorus*. It is shown that platycodin D disrupts the Hsp90–Cdc37 interaction and, subsequently, leads to the degradation of multiple Hsp90 client proteins without affecting Hsp90 ATPase activity [88]. However, its binding sites on the Hsp90–Cdc37 complex are unclear and need further studies.

**Apigenin** (**16**, Figure 4) is a natural product belonging to the flavone class and is abundant in common fruits and vegetables. Apigenin not only possesses anti-carcinogenic effects [89], but also plays important roles in pathogenesis of neurodegenerative diseases, such as AD, PD, multiple sclerosis, epilepsy, and stroke [90]. In multiple myeloma cells, apigenin disrupts the Hsp90–Cdc37–client complex, induces the degradation of multiple kinase clients, and decreases phosphorylation of Cdc37 [91]. Its binding sites on the Hsp90–Cdc37 complex also require investigation in further studies.

**18β-Glycyrrhetinic acid** is a pentacyclic triterpenoid found in *Glycyrrhiza glabra* L.(liquorice) roots. In 2018, a series of aminobenzothiazole derivatives of 18β-Glycyrrhetinic acid were designed and synthesized as Hsp90–Cdc37 disruptors with the ability to inhibit cell migration and drug-resistance [92]. Among them, **compound 17** (Figure 4) exhibits the most potent activity to disrupt the Hsp90–Cdc37 interaction (IC_50_, 0.14 μM). Its docking study suggested that compound 17 tightly binds to the active site of the Hsp90–Cdc37 complex and the small bulky and strongly electrophilic group of aminobenzothiazole side chains at its C-30 position is crucial for improving its activity.

##### Cdc37 Peptides

Another way to develop PPI inhibitors is to locate endogenous peptides at the binding epitopes of participating proteins. In 2015, the first small peptide, Pep-1 (Ac-KHFGMLRRWDD-NH2), was derived with high potency to disrupt the Hsp90–Cdc37 interaction [93]. The hot-spot Arg167 has been revealed as one of the most important binding determinants. Pep-1 not only occupies the Cdc37 binding site, but also interferes with Hsp90 ATPase activity. Later, more peptides were designed for verification. In 2017, a shorter peptide, Pep-5 (Ac-HFGMLRR-NH2), was found, which exhibited more stable binding and better ligand properties than Pep-1 [94]. In a recent study, five new peptides (NYSVWDHIEVSDDLSKDGFSKSMVN, NYSVWDHIEVDDDLSKDGFSKSMVN, NYSVWDHIEVEDDLSKDGFSKSMVN, LSKDGFSKSMVN, and PSKDIFLKSMIN) were designed and synthesized with the ability to disrupt the Hsp90–Cdc37–Cdk4 complex in co-immunoprecipitation experiments [95]. The discovery of these peptides improved our understanding of the Hsp90–Cdc37 binding interface. However, they still need further optimization to improve their membrane permeability, proteolytic stability, and drug-like potential.

##### Small-Molecule Inhibitors

Several critical residues at the Hsp90–Cdc37 binding interface have been identified by Jiang et al. in 2010 [96]. The Split Renilla luciferase protein fragment-assisted complementation bioluminescence shows that mutations in Hsp90 (Q133A, F134A, and A121N) and mutations in Cdc37 (M164A, R167A, L205A, and Q208A) reduce the Hsp90/Cdc37 interaction by 70–95%, and mutations in Hsp90 (E47A and S113A) and a mutation in Cdc37 (A204E) decrease the Hsp90/Cdc37 interaction by 50%. This study provides the “hot-spots” at the Hsp90–Cdc37 binding interface to develop small-molecule inhibitors. Several small-molecule inhibitors targeting the Hsp90–Cdc37 interaction have been developed recently. However, their effects on neurodegeneration have not yet been investigated.

In 2017, Wang et al., identified small molecules inhibiting the Hsp90–Cdc37 interaction by utilizing a structure-based virtual screening workflow, derivatives synthesis, and their biological evaluation [97]. The chemical databases (Specs and NCI database) with 500,000 molecules were screened by pharmacophore and cross-docking filtrations. After that, 31 compounds were obtained and purchased for further in vitro identification, including homogeneous time-resolved fluorescence assay, ATPase inhibition assay, fluorescence polarization assay, and direct binding assay (biolayer interferometry). Finally, **VS-8** (**18**, Figure 5) reveals moderate binding ability to Hsp90 (*K*_d_, 80 μM) and disrupts the Hsp90–Cdc37 interaction (IC_50,_ 77 μM) without effects on Hsp90 ATPase activity. To enhance the potency, 16 derivatives were designed and synthesized. Among them, **compound 19**, with N-methylpyrazole substitution and the central linker of two alkyl carbons, exhibits better binding capacity (*K*_d_, 40 μM), a more promising inhibitory effect (IC_50_, 27 μM), and preferable antiproliferative activity against multiple cancer cell lines.

In 2018, Chen et al. found that **DCZ3112 (20, Figure 5),** a novel derivative of triazine, directly binds to the NTD of Hsp90, inhibits the Hsp90–Cdc37 interaction without inhibiting ATPase activity, and leads to the degradation of Hsp90 client proteins [98]. DCZ3112 predominantly acts in HER2-positive breast cancer, exerts synergistic effects when applied in combination with anti-HER2 antibodies, and overcomes trastuzumab resistance [98].

In 2019, Wang et al. identified polar interactions between Arg167 on Cdc37 and Glu47 and Gln133 on Hsp90 as the most important binding determinants in recognition during the dynamic cycle of the Hsp90–Cdc37 interaction [99]. Then, they designed a screening workflow that identified **compound 21** (Figure 5), which has a moderate binding affinity to Hsp90. Based on the hit compound 21, **DDO-5936** (**22**) was identified as an active inhibitor disrupting the Hsp90–Cdc37 interaction, with no effects on Hsp90 ATPase activity. Although they failed to obtain the co-crystal structure of DDO-5936–Hsp90, the NMR detection and mutagenesis validation demonstrated that DDO-5936 specifically binds to the Glu47 on Hsp90 NTD to block the critical interaction between Hsp90 and Cdc37. In addition, inhibition of the Hsp90–Cdc37 complex by DDO-5936 results in downregulation of cyclin-dependent kinase 4 and consequent inhibition of cell proliferation through Cdc37-dependent cell cycle arrest, as well as *in vivo* antitumor potency in a xenograft model [99]. Later, the same group developed **compound 23** with improved binding affinity and antiproliferative activity, preferable stability in plasma and microsomes, and oral efficacy *in vivo*, compared with DDO-5936 [100]. Based on the binding mode of compound 21, they recently discovered a hydrophobic pocket centered on Phe213 of Hsp90 that contributes to the binding affinity of Hsp90–Cdc37 interaction inhibitors [101]. An optimum compound **DDO-5994** (**24**) was identified with an ideal binding on a Phe213 hydrophobic core. DDO-5994 has improved the binding affinity, antiproliferative activity, and antitumor potency in mice bearing HCT116 xenograft tumors.

**Niclosamide ethanolamine (NEN**, **25**, Figure 5) is an anthelmintic drug approved by the United States Food and Drug Administration for the treatment of parasitic infections. Several studies have shown that NEN can be used for the treatment of hepatocellular carcinoma [102], lipotoxicity [103], systemic lupus erythematosus [104], and artery constriction [105]. Chen et al. found NEN inhibits multiple kinases that are regulated by the Hsp90–Cdc37 complex, such as AKT, EGFR, STAT3, LRP6, and Raf family [102]. Using purified recombinant Hsp90 and Cdc37 in ELISA and co-immunoprecipitation assays, they found that NEN disrupts the Hsp90–Cdc37 interaction. Their results also showed that NEN binds to Cdc37 in pull-down assay and thermal shift assay, but its specific binding site on Cdc37 is unknown.

To identify novel Hsp90–Cdc37 interaction inhibitors, Siddiqui et al. recently established a mammalian cell lysate-based, medium-throughput amenable split *Renilla* luciferase assay, which employs N-terminal and C-terminal fragments of Renilla luciferase fused to full-length human Hsp90 and Cdc37, respectively [106]. Later, the same group screened more than 120,000 compounds via an FW-04-806 (conglobatin A)-based pharmacophore model and molecular docking, and confirmed the hits’ in vitro effects using their formerly established mammalian cell lysate-based split *Renilla* luciferase assay [85]. The active **compounds 26** and **27** were identified with K-Ras selectivity. Both compounds potently decrease the Hsp90 client protein levels without affecting Hsp90 ATPase activity, and inhibit the cancer cell proliferation and microtumor formation.

#### 3.2.2. Hsp90–Aha1 Interaction

Aha1 accelerates Hsp90 ATPase activity and has two domains, an NTD and a CTD, connected by a flexible linker [107,108]. In 2004, Meyer et al. reported a fragment-based crystal structure of Aha1 NTD in complex with Hsp90 MD that provides a model for Aha1 recruitment to Hsp90 [109]. In 2010, Koulov et al. have demonstrated that Aha1 NTD and CTD cooperatively bind across the dimer interface of Hsp90 to modulate the ATP hydrolysis cycle and Aha1 CTD binds to Hsp90 NTD, promoting ATP hydrolysis by stabilization of the N-terminal dimer interface [110]. At the same time, Retzlaff et al. also proved that acceleration of the Hsp90 ATPase cycle requires the interaction of both Aha1 NTD and CTD in a cooperative manner with both Hsp90 NTD and MD in an asymmetric activation mechanism [111]. In 2020, Liu et al. revealed that the Hsp90–Aha1 complex has six different states by cryo-electron microscopy [112]. Combining with previous data, they proposed a multistep activation model: Aha1 is firstly recruited to Hsp90 through interactions between Aha1 NTD and Hsp90 MD; then Aha1 CTD binds to Hsp90, which leads to a structural transition of Hsp90 from its open state to a semi-closed state and undocks Hsp90 NTD from Hsp90 MD; in the presence of ATP, Aha1 CTD rearranges its binding interface with Hsp90 MD, stabilizing a fully closed state followed by Hsp90 NTD dimerization; finally, Aha1 NTD tilts up to interact with dimerized Hsp90 NTD and facilitates ATP hydrolysis [112,113].

Aha1 has an important role in AD tauopathy. Aha1 increased tau fibril formation in the presence of Hsp90 *in vitro*. Overexpression of Aha1 in the rTg4510 tau transgenic mouse model increased neurotoxic oligomeric and insoluble tau accumulation, leading to both neuron loss and cognitive deficits [37]. Moreover, overexpression of Aha1 in aged wild-type mice impaired associative learning and promoted tau phosphorylation [38]. Therefore, small molecules inhibiting Hsp90–Aha1 PPI can be developed for the treatment of AD.

**Figure 6:**
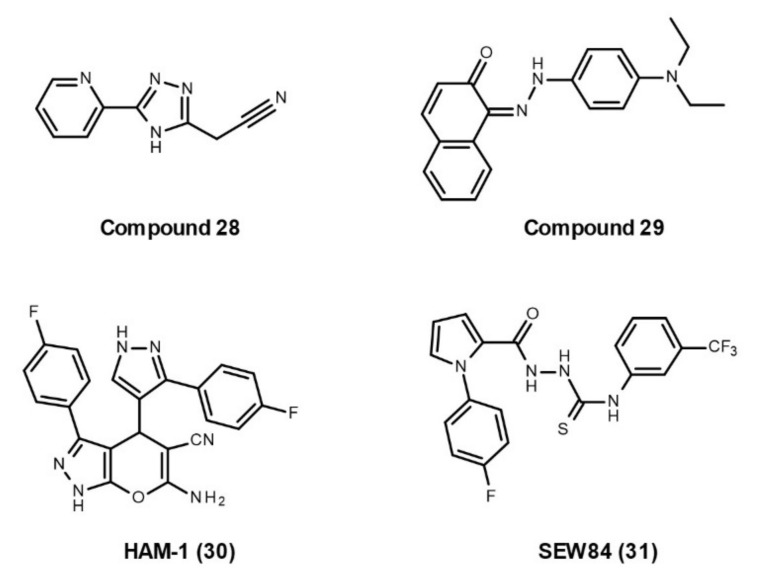
Developed small molecules with the ability to disrupt Hsp90–Aha1 interactions.

In 2017, Ihrig et al. first screened a collection of 14,400 drug-like compounds for Hsp90–Aha1 PPI inhibitors by AlphaScreen assay, and identified two drug-like inhibitors, **compounds 28** and **29** (Figure 6), that show positive effects on cystic fibrosis [114]. In the same year, Stiegler et al. screened about 15,000 chemical compounds by resonance energy transfer assay and selected six inhibitors with promising effects on the Hsp90–Aha1 interaction [115]. The most effective inhibitor, **HAM-1** **(30, Figure 6),** specifically prevents the interaction between Hsp90 NTD and Aha1 CTD, without dissociating the binding of Hsp90 MD and Aha1 NTD. Thus, HAM-1 does not change Aha1 affinity, but can abrogate the Aha1-induced ATPase stimulation of Hsp90 [115]. In 2020, Singh et al. built a modified quinaldine red-based high-throughput assay to screen compounds that inhibit Aha1-stimulated Hsp90 ATPase activity [116]. They identified a novel inhibitor of the Aha1-stimulated Hsp90 ATPase activity, **SEW84** (**31**, Figure 6), and revealed that it binds to the CTD of Aha1 to weaken its asymmetric binding to Hsp90. However, SEW84 does not affect the basal ATPase activity of Hsp90, avoiding the toxic effects of common Hsp90 inhibitors. Importantly, SEW84 reduces the tau phosphorylation in HEK293 cells expressing RFP-tagged 0N4R-tau, primary rat cortical neurons expressing endogenous WT-tau, and cultured brain slices from rTg4510 transgenic mouse [116]. SEW84 needs further *in vivo* experiments to verify its effect on tauopathies.

#### 3.2.3. Hsp90–p23 Interaction

Co-chaperone p23 preferentially interacts with Hsp90 and stabilizes the closed 2 state of Hsp90 [117]. The crystal structure of p23 and Hsp90 shows that the folded NTD of p23 binds to the junction of two NTDs of Hsp90. Another identified interaction site is located in the MD of Hsp90, which stabilizes the interaction with p23 [118,119]. The interaction between Hsp90 and p23 leads to the inhibition of Hsp90 ATPase activity [107,120,121]; that is, p23 either inhibits the hydrolysis process or impedes the subsequent release of ADP and Pi, and hence the re-opening of Hsp90 [122]. There is some evidence showing that p23 has an important role in AD and PD. Knockdown of p23 via siRNA transfection reduces both total and phosphorylated tau levels [39]. It is also proposed that the Hsp90–p23 complex contributes to neurotoxicity in PD. Mitochondrial stress increases the association of the Hsp90–p23 complex and the enzyme prolyl hydroxylase domain 2 in cultured dopaminergic neurons, while p23 inhibition prevents mitochondrial stress-induced neurotoxicity [40]. Therefore, inhibition of the Hsp90–p23 complex can provide a new strategy for AD or PD treatment.

**Figure 7:**
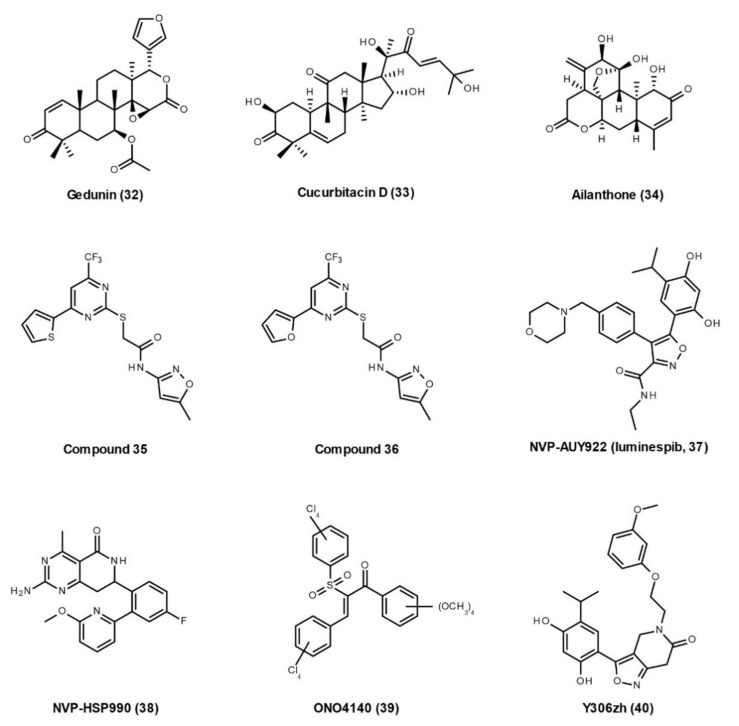
Developed small molecules with the ability to disrupt Hsp90–p23 interactions.

Celastrol (**8**), gedunin (**32**), cucurbitacin D (**33**), and ailanthone (**34**) are the four natural products that have been proved to inhibit Hsp90–p23 PPIs (Figure 7). Contrary to the former results [69], Chadli et al. reported that **celastrol** (**8**) can disrupt the Hsp90–p23 complex by altering the structure of p23, causing it to polymerize into amyloid-like fibrils [123]. **Gedunin** (**32**) is a tetranortriterpenoid natural product isolated from the Indian neem tree. Patwardhan et al. revealed that gedunin inhibits the p23 chaperoning activity, blocks its cellular interaction with Hsp90, prevents glucocorticoid receptor nuclear localization, and interferes with p23-mediated gene regulation [124]. Using molecular docking and mutational analysis, they identified three amino acids (Thr90, Ala94, and Lys95) that mediate noncovalent interactions of p23 with gedunin. In human breast and cervical cancer cell lines, gedunin induces apoptotic cell death and caspase-7-dependent cleavage of p23. Gedunin has been shown to have a potential therapeutic role in neurodegenerative diseases. It enhances tau degradation [125], inhibits oligomeric Aβ-induced microglia activation [126], and degrades the abnormal mutant huntingtin (HTT) aggregates and intranuclear inclusions in cells from HD patients [127]. Deoxygedunin also protects nigrostriatal dopaminergic neurons and improves the behavioral performance in PD animal models [128]. However, these studies do not evaluate the effects of gedunin on Hsp90-p23 PPIs in these neurodegenerative conditions. The underlying link between gedunin’s neuroprotective effects and its inhibition of Hsp90–p23 PPIs need to be investigated in the future. **Cucurbitacin D** **(33)** is an active component in *Cucurbita texana*. Co-immunoprecipitation showed that cucurbitacin D disrupts interactions between Hsp90 and two co-chaperones, p23 and Cdc37, in MCF7 cell lysates [129]. In 2016, He et al. identified a natural compound, **ailanthone** (**34**), that prevents Hsp90–p23 PPIs and decreases the interaction between chaperones and the androgen receptor (AR) followed by ubiquitin/proteasome-mediated degradation of AR as well as other p23 clients [130]. Ailanthone does not interact with Hsp90, but directly binds to p23 on the surface formed by Ser100, Val101, Lys95, Arg93, Pro87, and Trp8. Ailanthone not only blocks tumor growth and metastasis of castration-resistant prostate cancer, but also possesses favourable drug-like properties such as good bioavailability, high solubility, lack of CYP inhibition, and low hepatotoxicity [130]. Its effects on neurodegeneration need to be studied later.

In addition, Chan et al. developed a dual luciferase (Renilla and Firefly) reporter system for high-throughput screening and identifying inhibitors of Hsp90–p23 interactions [131]. Using this method, they identified a potent compound, *N*-(5-methylisoxazol-3-yl)-2-[4-(thiophen-2-yl)-6-(trifluoromethyl)pyrimidin-2-ylthio]acetamide (**35**, Figure 7), that inhibits Hsp90–p23 bioluminescence imaging signals and leads to the degradation of Hsp90 clients. They also performed a structural activity relationship study with 62 analogs of compound 35, and identified **compound 36** as the lead compound that outperformed compound 35 in inhibiting Hsp90–p23 interactions. Some Hsp90 inhibitors with anticancer activities also induce Hsp90–p23 dissociation, such as **NVP-AUY922** (**luminespib**, **37**), **NVP-HSP990** (**38**), **ONO4140** (**39**), and **Y306zh** (**40**) [132,133,134,135]. Y306zh binds to the NTD of Hsp90, causes ATP to be incapable of attaching to Hsp90 NTD, and thus disrupts Hsp90–p23 PPIs [135].

#### 3.2.4. Hsp90–Hop Interaction

Several Hsp90 co-chaperones contain the TPR domain that mediates the binding to the conserved MEEVD pentapeptide at the CTD of Hsp90 [136]. One of the best characterized TPR-containing co-chaperones is Hop, which facilitates unfolded client proteins transferred from Hsp70 to Hsp90 for maturation. Hop contains three TPR domains, TPR1, TPR2A, and TPR2B. Its TPR1 and TPR2B domains are responsible for Hsp70 binding, while the TPR2A domain is for Hsp90 binding [137,138]. The TPR2A and TPR2B domains of Hop also interact with the MD of Hsp90 [138].

Hop is important in HD, AD, and prion diseases [139]. Hop overexpression in yeast inhibits the toxicity of HTT with 103Q glutamine stretch (HTT103Q) and reorganizes small HTT103Q foci into larger assemblies [41]. Downregulation of Hop enhances tau toxicity in a fly model of tauopathy [42]. Secreted Hop binds to the cellular prion protein (PrP^C^) and promotes calcium influx through α7 nicotinic acetylcholine receptors (α7nAChRs), which modulates neuronal differentiation and survival [43]. As the PrP^C^ ligand, Hop also inhibits Aβ oligomers’ binding to PrP^C^ and prevents synaptic loss, neuronal death, and depression of long-term potentiation induced by Aβ oligomers [44]. The TPR1 and TPR2A of Hop contribute to the binding of C-terminal of PrP^C^ and can directly inhibit both Aβ oligomers’ binding to PrP^C^ and neuronal toxicity [140]. Excess Hsp90 can disrupt the Hop–PrP^C^ interaction by interfering with the TPR2A domain of Hop and blocks Hop neuroprotective functions [140]. Therefore, disrupting the Hsp90–Hop interaction can promote the degradation of Hsp90 clients and provide more Hop to bind with PrP^C^, leading to a new therapeutic strategy for neurodegenerative disease.

**Figure 8:**
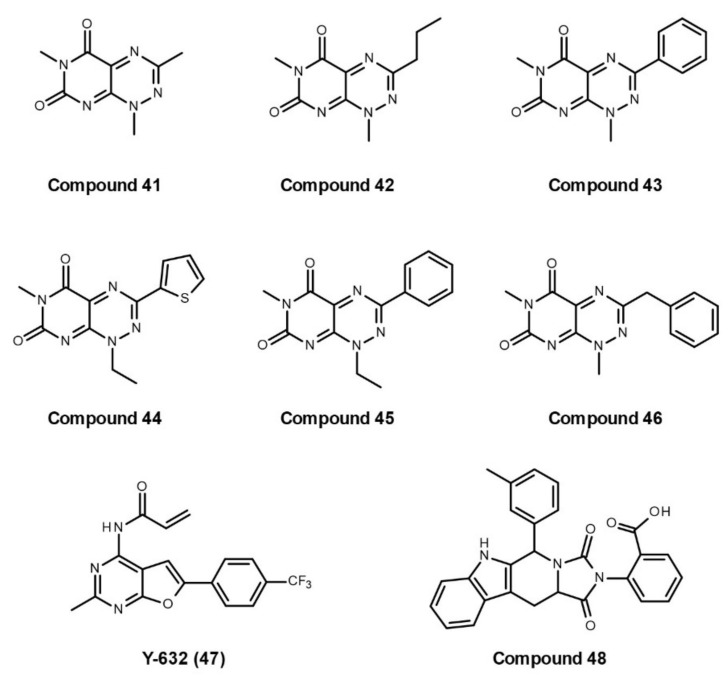
Developed small molecules with the ability to disrupt Hsp90–Hop interactions.

In 2008, Yi and Regan first screened more than 97,000 compounds by AlphaScreen assay to identify a new class of small molecules that inhibit the interaction of the C-terminal peptide of Hsp90 and the TPR2A domain of Hop [141]. Following structure clustering analysis, competition confirmation, counter screen for false positives, fluorescence polarization assay, and isothermal titration calorimetry, they have identified six active compounds, **41–46** (Figure 8), with a 7-azapteridine ring system that binds at a key position on the TPR2A interaction interface. These compounds can reduce the levels of the Hsp90-dependent client protein HER2 in human breast cancer cell lines without inducing Hsp70 overexpression. Later, the same group characterized the anticancer activity of **compound 42** (Figure 8) and showed that this compound is effective in killing different breast cancer cell lines including the highly metastatic MDA-MB-231 [142].

In 2011, Horibe et al. designed the hybrid Antp-TPR peptide (KAYARIGNSYFK) based on the structure of the Hsp90–TPR2A complex [143]. This peptide includes the conserved amino acids Lys301 and Arg305 that donate hydrogen bonds to the Hsp90 C-terminal region. The surface plasmon resonance experiments showed that the hybrid Antp-TPR peptide specifically inhibits the interaction of Hsp90 with Hop, rather than the interaction of Hsp70 with Hop and the interaction of Hsp90 with FKBP5 or PP5. They also proved the antitumor activity of this peptide both in various cancer cell lines and in a xenograft model of human pancreatic cancer in mice. Later, another peptide PEP73 (INSAYKLKYARG) was designed based on the in silico docking of Hsp90α and Hop, but its inhibition effects need to be further verified [144].

In 2016, Wang et al. found a pyrimidine derivative, **Y-632** (**47**, Figure 8), as a novel inhibitor disrupting the interaction between Hsp90 and Hop [145]. Y-632 neither binds to Hsp90 nor inhibits Hsp90 ATPase activity. It inhibits Hsp90 function by intracellular thiol oxidation, thereby disturbing the Hsp90–Hop interaction. Y-632 induces the degradation of diverse Hsp90 client proteins through the ubiquitin-proteasome pathway and can efficiently overcome imatinib resistance mediated by Bcr-Abl point mutations.

Recently, Darby et al. mimicked key native “carboxylate clamp” interactions between Hsp90 and its TPR co-chaperones and designed several compounds that block the interaction between Hop TPR2A and Hsp90 C-terminal MEEVD peptide after the failure of AlphaScreen high-throughput screening [146]. The binding of these compounds to the Hop TPR2A domain was confirmed by mapping ^1^H-^15^N HSQC chemical shift perturbations to their new reported NMR solution-state structure of Hop TPR2A. Co-immunoprecipitation was also used to prove the Hsp90–Hop PPI inhibition effects of a selected compound, **48** (Figure 8), in human cancer cells. However, these compounds have relatively low potency to disrupt Hsp90–Hop PPI. Much effort is still required to overcome the challenge for finding efficient small-molecule ligands disrupting the Hop TPR2A–Hsp90 MEEVD interface.

#### 3.2.5. Hsp90 and Other TPR Co-Chaperones Interactions

In addition to the aforementioned Hop, there are several other important TPR containing co-chaperones of Hsp90, such as PP5, C terminus of Hsp70-interacting protein (CHIP), FKBP 51, and FKBP52. PP5 contains the phosphatase domain in the C-terminal region and three consecutive TPR domains in the N-terminal region for PPIs. Its TPR domains interact with its extreme C-terminal alpha J helix (αJ) in the auto-inhibited state, which blocks substrate access to the catalytic groove [147]. This auto-inhibition can be broken by interacting the PP5 TPR domains with PP5 activators such as chaperones Hsp70 and Hsp90. PP5 can directly dephosphorylate Hsp90, which modulates Hsp90 conformational cycle and client maturation [148,149]. Additionally, binding of PP5 to the Hsp90–Cdc37–kinase heterocomplex leads to Cdc37 dephosphorylation and subsequent release of Cdc37 and mature kinase. CHIP contains an N terminal TPR domain together with a U-box domain linked via a long helical region [150]. CHIP binds to both Hsp70 and Hsp90 via its TPR domain and interacts with the proteasome by acting as an E3 ligase using its U-box domain, which effectively crosslinks the chaperones to the ubiquitin-proteasome system for substrate degradation [151]. FKBP51 and FKBP52 share high homology and the same domain structure. They contain the PPIase domain (FK1), as well as FK2 and TPR domains. With the PPIase activity, they were classified as immunophilins owing to their tight binding to the immunosuppressants FK506 and rapamycin. FKBP51 and FKBP52 have been shown to participate in the Hsp90–steroid receptor complex and regulate the progression of the Hsp90 conformational cycle [152].

##### Hsp90 TPR Co-Chaperones in Neurodegenerative Diseases

These TPR co-chaperones have important roles in neurodegenerative diseases. PP5 is a serine/threonine protein phosphatase that can dephosphorylate tau at AD-associated abnormal phosphorylation sites and its activity decreases in AD neocortex [45]. PP5 also protects primary rat cortical neurons from cell death induced by Aβ [46]. Acting as an E3 ligase for ubiquitination degradation, CHIP has been involved in several neurodegenerative diseases where protein aggregates are a hallmark, such as AD, PD, and HD. Specifically, the Hsp90–CHIP complex is essential for the degradation of phosphorylated tau in AD, and the deletion of CHIP in mice leads to the accumulation of hyperphosphorylated and caspase-3 cleaved tau species [39,47]. In PD, CHIP reduces α-synuclein oligomerization and mediates α-synuclein degradation [48,49]. The overexpression of CHIP in cell culture inhibits α-synuclein inclusion formation and reduces α-synuclein levels [49]. CHIP also reduces the uptake of α-synuclein fibrils by Neuro-2a cells that interfere with the propagation of pathogenic α-synuclein assemblies [50]. Moreover, CHIP binds, ubiquitinates, and promotes the ubiquitin proteasomal degradation of LRRK2 [51,52]. Overexpression of CHIP protects against and knockdown of CHIP exacerbates the toxicity of mutant LRRK2 [51]. In HD, transient overexpression of CHIP increases the ubiquitination and the rate of degradation of polyglutamine-expanded HTT or ataxin-3 [53]. CHIP suppresses polyglutamine aggregation and toxicity, and its haploinsufficiency markedly accelerates disease phenotype in an HD transgenic mouse model [54]. FKBP51, in complex with Hsp90, enhances the production of tau oligomers and prevents tau degradation by the 20S proteasome [55]. The levels of FKBP51 increase with age in the mouse brain, and AD patients have a higher expression of FKBP51 than age-matched controls [55,56]. FKBP51 is also involved in Pink1′s regulation of AKT on neuronal survival [57]. Moreover, downregulation of FKBP51 reduces mutant HTT levels in HD models both in vitro and *in vivo* [58]. The function of FKBP52 in AD is complicated. FKBP52 induces aggregation of multiple tau species in vitro [59,60,61]. Overexpression of FKBP52 in the hippocampus leads to cognitive impairments and neurotoxicity in aged wild-type mice and rTg4510 transgenic mice [38,62]. However, FKBP52 levels are abnormally low in the frontal cortex of AD brains, as compared with controls [63]. It is suggested that this abnormal decrease of FKBP52 levels in the AD brain hinders autophagy efficiency and contributes to tau pathology [153]. In *Drosophila*, which expresses Aβ peptides, downregulation of FKBP52 potentiates Aβ toxicity, while FKBP52 overexpression suppresses Aβ toxicity and increases the lifespan [64]. Thus, further studies are required to elucidate the roles of FKBP52 in the progression of AD. In PD, FKBP52 overexpression accelerates α-synuclein aggregation and neuronal cell death, whereas knockdown of FKBP52 reduces α-synuclein aggregation and prevents cell death [65]. Moreover, FKBP52 can generate immune responses to α-synuclein-based immunizations in mice [66]. Therefore, modulating the interaction between Hsp90 and these TPR co-chaperones can represent a therapeutic strategy for neurodegenerative diseases.

##### Hsp90–TPR Co-Chaperone Interaction Inhibitors

**Figure 9:**
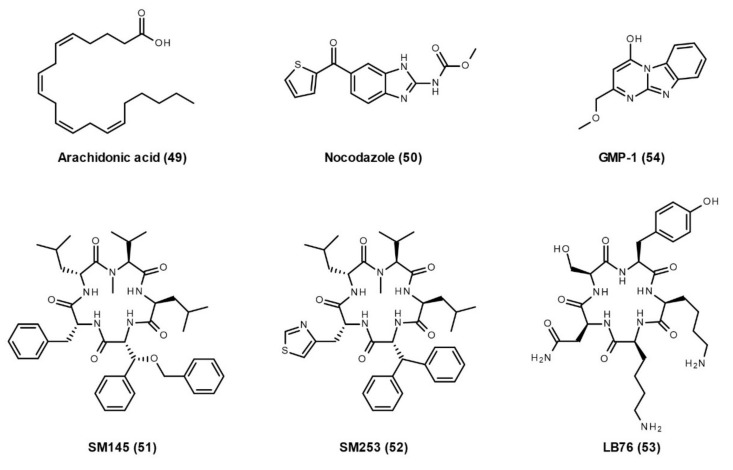
Developed small molecules with the ability to disrupt interactions between Hsp90 and TPR co-chaperones.

Currently, there are only a few publications reporting the inhibitors of the interactions between Hsp90 and TPR co-chaperones. In cell-based studies, **arachidonic acid** (**49**, Figure 9) or **nocodazole** (**50**, Figure 9) can inhibit the interaction of Hsp90 with FLAG-tagged PP5 via the TPR domain of PP5 and increase the phosphatase activity of PP5 [154]. As a derivative of the natural product Sansalvamide A, **SM145** (**51**, Figure 9) binds between the NTD and MD of Hsp90 and allosterically disrupts the interaction between Hsp90 and all tested TPR co-chaperones, such as Hop, FKBP51, FKBP52, FKBP38, and cyclophilin 40 (Cyp40) [155,156]. It is speculated that SM145 induces or stabilizes an Hsp90 conformation that hinders access to MEEVD residues where TPR domains interact [155]. SM145 also induces a caspase-3 dependent apoptotic event and leads to a decrease in hormone receptor protein levels without triggering the heat shock response [155,156]. However, SM145 has poor overall synthetic yields and low solubility. Later, a new analogue, **SM253** (**52**, Figure 9), was synthesized with relatively high overall yields and good aqueous solubility [157]. Another group designed a new compound, **LB76** (**53**, Figure 9), that binds directly to the CTD of Hsp90 and interacts with its MEEVD residues, thereby disrupting the interactions between Hsp90 and TPR co-chaperones, such as Hop, Cyp40, FKBP51, and FKBP38 [158]. LB27 blocked the interaction between Hsp90 CTD and TPR co-chaperones with different IC_50_ values: 4 µM for Hop, 7.2 µM for Cyp40, 48 µM for FKBP38, and 100 µM for FKBP51. This work provides the possibility to design small molecules that selectively inhibit the interaction between Hsp90 and TPR proteins. In addition, our group has identified a novel compound, **GMP-1** (**54**, Figure 9), that disrupts interactions between Hsp90/70 and the TPR protein, mitochondrial protein import receptor Tom70 [159]. Treatment with GMP-1 demonstrated its neuroprotective effects in mouse and *Drosophila* models of AD.

##### Hsp90/Hsp70 Interaction with Dicarboxylate Clamp TPR (dcTPR) Co-Chaperones

**Figure 10:**
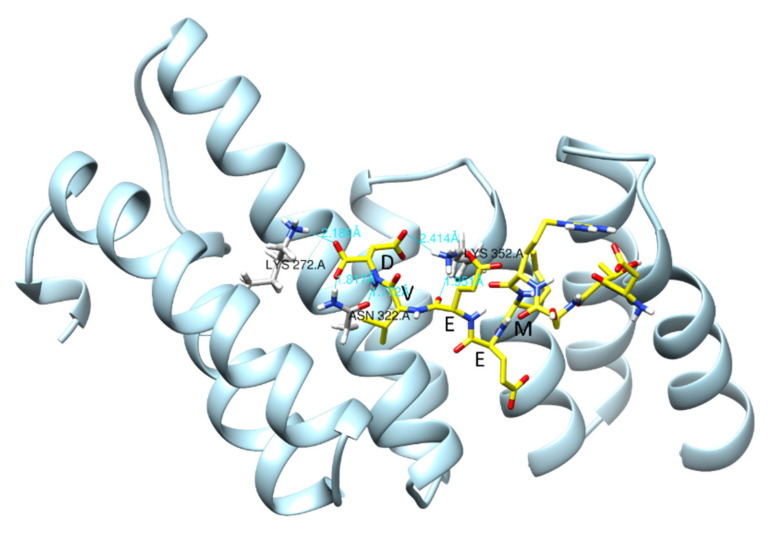
Dicarboxylate clamp mechanism in the interaction of the TPR domain of FKBP51 (shown as ribbon) and C-terminal MEEVD peptide of Hsp90 (shown as bonds with carbons in yellow colour). The two carboxylic acid groups of terminal aspartate of Hsp90 form hydrogen bonds with Lys272 at a distance of 2.18 Å, with Asn322 at a distance of 1.817 Å, and with Lys352 at a distance of 2.414 Å. The figure is generated using PDB id: 5NJX in UCSF-Chimera [163]. For clarity purposes, the FK1 and FK2 domains of FKBP51 are omitted.

Molecular co-chaperones containing the TPR domain interact with Hsp90/Hsp70 via the formation of a dicarboxylate clamp between side chains of the residues in the TPR domain (dcTPR) and two carboxylic acid groups of the C-terminal aspartate or glutamate of the Hsp90/Hsp70 [160]. The residues involved in dicarboxylate clamp formation are conserved in most of the TPR-Hsp90/Hsp70 interactions [161]. In the case of the interaction between the dcTPR domain of FKBP51 and Hsp90, residues Lys272 and Lys352 are involved in the formation of dicarboxylate clamp with the C-terminal aspartate of Hsp90 through hydrogen bonding and salt bridges (Figure 10). We have recently reported a conservational profile of several dcTPR domain-containing proteins (HOP, FKBP52, CHIP, AIP, FKBP38, FKBP51, and Tah1), which supports the conservative nature of dicarboxylate clamp forming residues. It was found that Lys272, Lys352, Asn322, Glu273, and Lys329 were conserved throughout these proteins, out of which Lys272 and Lys352 are the two residues involved in the formation of the dicarboxylate clamp [161]. In another study, we have identified dcTPR interacting proteins having an acidic C-terminal domain similar to Hsp90/Hsp70 [162].

The development of ligands targeting the dcTPR domain for inhibition of Hsp90/co-chaperone interaction is considered as an alternate strategy for drug development against these targets. However, there are some limitations such as the charged nature of compounds with which to compete and disruption of the dicarboxylate clamp along a poor pharmacokinetic profile of such compounds [164]. Another challenge in the development of these compounds is the selective inhibition of Hsp90/co-chaperone interactions because of its highly conservative nature. However, our lab is continuing the development of ligands targeting the dcTPR domain using a combined in silico and structural biology approach. To expedite the identification of hits as inhibitors of the dcTPR-Hsp90/Hsp70 interaction, we have developed and validated a high throughput AlphaScreen assay to screen compound libraries [165]. We have obtained several selective and potent inhibitors of the Hsp90–FKBP51 interaction (unpublished results).

## 4. Conclusions

For most neurodegenerative disorders, only symptomatic treatment is available. There is an urgent need for the development of new therapeutics with disease-modifying properties. Because protein misfolding and aggregation is a major molecular hallmark of these brain disorders, strategies to modulate Hsp70/Hsp90 molecular chaperone networks are particularly attractive. In addition to general difficulties associated with the development of central nervous system (CNS) drugs, life-long treatment will set an additional hurdle for such molecules in terms of their potential toxicity. Therefore, modulation of the Hsp70/Hsp90 network by interference with small molecules affecting interactions between chaperones and their specific co-chaperones can change neuronal proteostasis and ultimately modify the course of the disease.

Modulation of PPIs through small molecules is generally considered difficult for the following reasons. PPIs often occur at the protein surface interfaces of a flat shape and large area (1500–3000 Å^2^). This makes it difficult for small molecules to bind with high affinity breaking selective PPIs. Moreover, drugs acting on PPIs tend to have a higher molecular weight (>400 Da) as compared with traditional small molecule drugs (200–500 Da), making it challenging to meet the criteria like Lipinski’s “rule of 5” required for CNS drugs.

With regards to chaperone/co-chaperone interactions, two types of PPIs can be observed: PPIs where the interaction area is large, and PPI inhibitors that would allosterically interact with their targets. For example, only allosteric inhibitors of Hsp70 interactions with J-proteins and NEFs have been developed so far. Such PPI inhibitors would lack selectivity towards particular co-chaperones; however, developing Hsp70 isoform-specific molecules can provide sufficient selectivity for modulation of biological processes. On the other side, PPIs of dcTPR co-chaperones with Hsp70/Hsp90 are mediated by a short C-terminal -EEVD peptide. The -EEVD peptide-binding groove of dcTPR proteins has an area of 650–750 Å^2^ [137]. Moreover, several X-ray structures of dcTPR proteins with or without C-terminal peptide of Hsp90/Hsp70 have been solved. This allows to implement computer-aided drug design to obtain small inhibitors of chaperone/co-chaperone PPIs. Again, the selectivity of the inhibitors is a major issue. The binding site for the -EEVD peptide has a similar tertiary structure and conserved amino acids responsible for the interaction, but the overall homology between different dcTPR domains of co-chaperones is below 50% [137]. Theoretically, reasonable specificity for small molecules (modulators) competing for -EEVD peptide can be achieved, as reported for the FKBP51/FKBP52 pair by our group [165]. Most likely, however, it will be impossible to achieve high specificity towards each member of the dcTPR protein family, which, in humans, consists of more than 20 proteins.

Finally, modulation of the molecular chaperone network by inhibition of particular chaperone/co-chaperone interactions with small molecules is an attractive strategy to treat neurodegenerative disorders. However, the development of such PPIs as CNS drugs poses great challenges that need to be resolved at the level of drug potency, selectivity, toxicity, and CNS permeability.

## Figures and Tables

**Table 1:** Hsp90 co-chaperones, their regulation of Hsp90 function, and involvement in processes related to neurodegeneration.

Co-Chaperone	Full Name	Interacting Domain in Co-chaperone	Binding Site in HSP90	Function	Disease	Cellular Processes
CDC37	Cell division cycle 37	MD, NTD	NTD, MD	Prevents closure of the “lid” in HSP90;Specific for maturation of kinases	AD, PD, ALS, FTLD	Stabilizes tau via Hsp90 and regulates the stability of distinct tau kinases, specifically Cdk5 and Akt [31]; Preserves TDP-43 [32]; Its client kinases include DYRK1A [33] Stabilizes LRRK2 [34]; Stabilizes PINK1 and influences its subcellular distribution [35,36]
Aha1	Activator of Hsp90 ATPase homolog 1	NTD, CTD	NTD, MD	Stimulates ATPase activity of HSP90	AD	Increases tau fibril formation, Aha1 overexpression in rTg4510 mouse increases tau accumulation, leading to both neuron loss and cognitive deficits [37]. Aha1 overexpression in aged wild-type mice impairs associative learning and promotes tau phosphorylation [38].
p23 (Sba1 in yeast)	Co-chaperone p23	NTD	NTD, MD	Stabilizes the HSP90 closed 2 state; Inhibits Hsp90 ATPase activity	AD, PD	Knockdown of p23 reduces both total and phosphorylated tau levels [39]. Contributes to neurotoxicity in PD [40].
Hop (Sti1)	Hsp70-Hsp90 organizing protein (stress-inducible phosphoprotein 1)	TPR	CTD, MD	Transfers clients from Hsp70 to Hsp90; Inhibits Hsp90 ATPase activity	HD, AD, Prion diseases	Hop overexpression in yeast inhibits the toxicity of HTT103Q and reorganizes small HTT103Q foci into larger assemblies [41]. Hop downregulation enhances tau toxicity in the fly model of tauopathy [42]. Binds to PrPC and promotes calcium influx through α7nAChRs [43]. Inhibits Aβ oligomers’ binding to PrPC and prevents synaptic loss, neuronal death, and depression of long-term potentiation induced by Aβ oligomers [44].
PP5 (Ppt1 in yeast)	Protein phosphatase 5	TPR	CTD	Dephosphorylates Hsp90; Dephosphorylates Cdc37	AD	Dephosphorylates tau and its activity decreases in AD neocortex [45]. Protects primary neuron death induced by Aβ [46].
CHIP	C terminus of Hsp70-interacting protein	TPR	CTD	E3 ubiquitin ligase	AD, PD, HD	Promotes the degradation of phosphorylated tau [39,47]. Reduces α-synuclein oligomerization and mediates α-synuclein degradation [48,49]. Reduces the uptake of α-synuclein fibrils by neuro-2a cells [50]. Promotes the degradation of LRRK2 [51,52]. CHIP overexpression promotes the degradation of polyglutamine-expanded HTT or ataxin-3 [53]. Suppresses polyglutamine aggregation and toxicity [54].
FKBP51	FK506 binding protein 51 kDa	TPR	CTD	Peptidyl-prolyl isomerase activity; Participates in Hsp90-steroid receptor complex; Generally regulates Hsp90 conformational cycle	AD, PD, HD	Enhances the production of tau oligomers and prevents tau degradation [55]. Increases with age in the mouse brain, and its expression is higher in AD patients [55,56]. Involved in Pink1′s regulation of AKT on neuronal survival [57]. FKBP51 downregulation reduces mutant HTT levels in HD models both in vitro and in vivo [58].
FKBP52	FK506 binding protein 52 kDa	TPR	CTD	Peptidyl-prolyl isomerase activity; Participates in Hsp90-steroid receptor complex; Generally regulates Hsp90 conformational cycle	AD, PD	Induces aggregation of multiple tau species in vitro [59,60,61]. FKBP52 overexpression in the hippocampus leads to cognitive impairments and neurotoxicity in aged wild-type mice and rTg4510 transgenic mice [38,62]. FKBP52 levels are abnormally low in the frontal cortex of AD brains [63]. Suppresses Aβ toxicity and increases the lifespan of *Drosophila*, which expresses Aβ peptides [64]. Accelerates α-synuclein aggregation and neuronal cell death [65]. Generates immune responses to α-synuclein-based immunizations in mice [66].

## Data Availability

Not applicable.
